# Simultaneous Dual Pathology in Lymph Node

**DOI:** 10.4084/MJHID.2014.036

**Published:** 2014-06-01

**Authors:** Prakas Kumar Mandal, Supriyo Sarkar, Malay Kumar Ghosh, Maitreyee Bhattacharyya

**Affiliations:** 1 Assistant Professor, Department of Hematology, Nilratan Sircar Medical College, Kolkata-700014, WB, India.; 3 Professor, Department of Hematology, Nilratan Sircar Medical College, Kolkata-700014, WB, India.; 2 Professor, Department of Respiratory Medicine, Nilratan Sircar Medical College, Kolkata-700014, WB, India.

**Dear Editor,**

We read with interest the article recently published in Mediterranean journal of Hematology and Infectious Diseases, in whom Fattorini et al. pointed out that two billion people worldwide are latently infected with Mycobacterium Tuberculosis (Mtb), with a 10% reactivating to active tuberculosis (TB) due to re-growth of non-replicating (dormant) Mtb residing in their tissues.^1^ The mechanism of reactivation is complex and can be attributed to the treatment with corticosteroids and/or any other condition affecting T cell function^2^,^3^. Here we report a case of concurrent TB lymphadenitis and diffuse large B-cell lymphoma (DLBCL). Tuberculous lymphadenitis is one of the common extra-pulmonary tuberculosis (TB); it is usually diagnosed by fine needle aspiration cytology (FNAC) which demonstrates caseating epithelioid granulomas and presence of acid fast bacilli (AFB). Majority of TB lymphadenitis respond to anti-TB therapy (ATT). However new lymph nodes may appear, or existing lymph nodes may increase in size during treatment in immune-suppressed as well as immune-competent patients.^4^ The most important cause of unresponsive lymphadenopathy is infection caused by atypical or drug resistant Mycobacteria. However rarely simultaneously double pathology of lymph node may be responsible for unresponsiveness to ATT extremely diverse pathology.

A 72 years old, non-smoker, non-alcoholic, hypertensive and non-diabetic female patient attended a private hospital with a history of low grade fever, cough and multiple swellings in the neck. Her peripheral blood counts, chest X-ray, was normal. FNAC from the cervical lymph node showed caseous necrosis and presence of acid fast bacilli (AFB) in Ziehl Neelsen stain. Her sputum was negative for AFB. ATT was started as per standard guideline. After one and half month of treatment, her condition deteriorated existing lymph nodes, appearance of new lymph nodes with persisting fever, progressively increasing size of in axillary and inguinal regions. Also, there was significant weight loss.

At this stage, she was referred to the Haematology department of our institute by the chest physician from the private hospital. She had pallor, bilateral pitting oedema, but no organomegaly. Respiratory examination showed right sided pleural effusion. Complete haemogram showed hemoglobin, 108 gm/l; total leucocyte count, 8.3×109/l with 65% neutrophil, 26% lymphocytes, 03% eosinophil, 06% monocyte; Platelets, 1.58×109/l, ESR–110mm in hour, Corrected reticulocyte count, 2.1% and Direct Coombs Test was negative. Serum chemistry was normal. Screening for viral markers HBsAg, Anti-HCV and anti-HIV-I &II were negative. Contrast enhanced computed tomography (CECT) of thorax and neck revealed bilateral enlargement (more evident on the right side) of cervical, mediastinal, axillary lymph nodes and pleural effusion. CECT of the abdomen including pelvis revealed multiple, enlarged retroperitoneal and bilateral inguinal lymph nodes. Pleural fluid cell count was 2500 × 109/l with the presence of atypical mononuclear cells, glucose 39 mg/dl, protein 3.2 g/l and ADA (adenosine deaminase) 28 IU/L. Cervical lymph node excisional biopsy revealed complete effacement of the lymph node architecture with presence of diffuse, monotonous large atypical lymphoid cells having irregular nuclear contour, vesicular nuclei and prominent multiple nucleoli suggestive of Non Hodgkin’s Lymphoma (NHL), diffuse, large cell type (Figure 1).

Immunohistochemistry (IHC) of the tumor cells expressed CD20, PAX-5, CD30 and negative for CD3, Alk-1, CD15, Cytokeratins. Trephine biopsy of bone marrow did not show any granuloma or lymphoid infiltration. Echocardiography revealed good left ventricular systolic function with mild diastolic dysfunction. Considering the available clinical, radiological and histopathological data, she was diagnosed as a case of DLBCL, Stage-IVB, international prognostic index (IPI) score-4(high risk) with concurrent tubercular lymphadenitis. She received six cycles of R-CHOP (Rituximab, Cyclophosphamide, Doxorubicin, Vincristine, Prednisolone) along with ATT. She achieved complete remission (CR) of both the diseases confirmed by negative whole body PET-CT and bone marrow biopsy, and biopsy from one persistent right axillary lymph node showed fibro-fatty changes without any evidence of TB or malignancy. TB lymphadenitis usually presents with multiple matted cervical lymph node enlargement with variegated consistency and sometimes with sinus formation; whereas, in lymphomas, lymph nodes are discrete and firm in consistency. Histopathological demonstration of granuloma is not necessary for diagnosis of TB lymphadenitis if FNAC demonstrates AFB. The diagnosis of NHL is confirmed by histopathological examination and immunohistochemistry.

In a country like India, both TB and NHL are common, but simultaneous occurrence of the diseases has not been reported before. NHL suppresses immune system directly or as a result of treatment. As lymphomas primarily involve the lympho-reticular system, they decrease cell mediated immunity.^5^ Depressed cell-mediated immunity can activate dormant bacilli in lymph nodes and thereby might cause active TB lymphadenitis.^3^ Various viruses like Epstein Barr virus, human herpes virus 8, human immunodeficiency virus has been implicated as possible etiologic factors of different types of NHL. The hypothesis in favor of these is i) viruses directly transforming lymphocytes or ii) causing profound depletion of CD4+ T-lymphocytes. NHL in many conditions has been found to be preceded by chronic inflammatory diseases.^5^ Association of Helicobacter pylori, campylobacter and Hepatitis C virus with NHL has already been well established.5 But till date, there is no evidence that Mycobacterium tuberculosis predisposes to NHL.

## Conclusion

Randomized study to compare CHOP chemotherapy plus rituximab with CHOP alone in elderly patients with diffuse large-B-cell lymphoma revealed that, addition of rituximab to the CHOP regimen increases the complete-response rate and prolongs event-free and overall survival in elderly patients, without clinically significant increase in toxicity.^7^ The present case achieved complete remission with R-CHOP regimen and at the same time the patient was also cured of TB with ATT. Surprisingly, TB did not response till NHL was taken care of, which is difficult to explain. TB is affected by cell-mediated immunity (T-cells) mainly, whereas DLBCL is primarily a B cell neoplasm. Though combination of TB lymphadenitis and Hodgkin’s lymphoma has been reported,^8^ the combination of TB lymphadenitis and NHL has not been signaled in the current literature. Our data do not permit to establish if TB precipitated NHL by chronic immune-stimulation or immunodepression present in NHL activated dormant TB bacilli in lymph nodes. A third possibility might be that, both the diseases occurred as a separate entity without any causal relationship. The case will give some impetus to think differently in cases of unresponsive lymphadenitis and raise a question whether histopathological examination of lymph nodes should be done despite the demonstration of AFB in FNAC

## Figures and Tables

**Figure 1:**
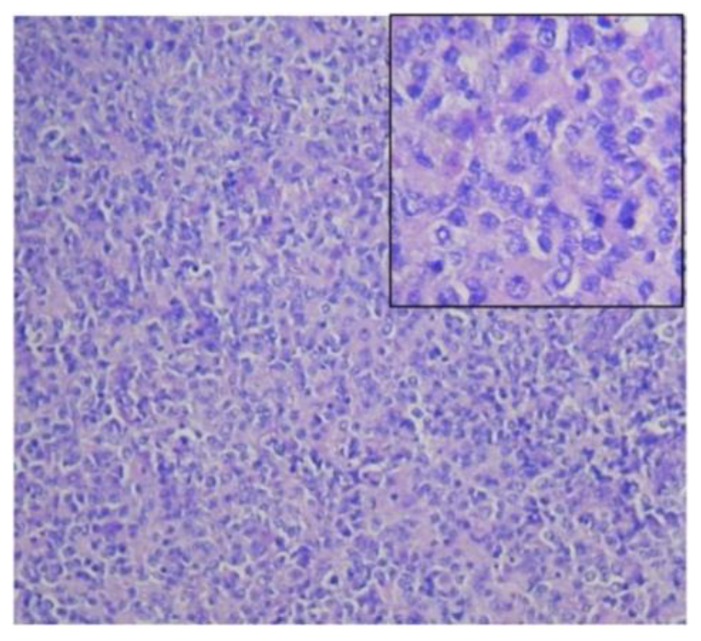
Cervical lymph node biopsy (hematoxylene and eosin stain) revealed histopathology of Non Hodgkin’s Lymphoma (NHL), diffuse, high grade, large cell type (inset- 400× magnification).
